# How Social Communications Influence Advertising Perception and Response in Online Communities?

**DOI:** 10.3389/fpsyg.2017.01349

**Published:** 2017-08-14

**Authors:** Fue Zeng, Ran Tao, Yanwu Yang, Tingting Xie

**Affiliations:** ^1^School of Economics and Management, Wuhan University Wuhan, China; ^2^Center for Marketing Research and Application, Wuhan University Wuhan, China; ^3^School of Management, Huazhong University of Science and Technology Wuhan, China; ^4^Department of Marketing, Hang Seng Management College Hong Kong, China

**Keywords:** online community, social bonds, advertising response, social communication, community advertising

## Abstract

This research aims to explore how social communications of online communities affect users’ perception and responses toward social media advertising. We developed a conceptual model based on the SBT, encapsulating 9 constructs and 10 hypothesis extracted from the extant social media advertising literature. Our research outcome proves that social communications can effectively boost users’ behaviors to be in accordance with an online social community, thus facilitate their acceptance and responses toward social media advertising, with users’ group intention as an intervening factor. From an operational standpoint, it’s an effective way to build and maintain social bonds between users and the community by boosting social communications, supporting fluent interpersonal communications. In addition, managers of an online community should elaborate on users’ group intentions to increase users’ advertising acceptance and response.

## Introduction

In the past several decades, plenty of successful social media advertising cases have increasingly attracted global firms, e.g., “Will it blend" by K-TEC (Briggs, 2009) and P&G’s “Smell Like a Man, Man" (P&G, 2010). Social media advertising is featured by several advantages such as the accurate targeting, ads co-creation between users and advertisers, multilateral interactions among communities, users, and advertisers. These fashions encourage advertisers to invest a large portion of advertising budget into the burgeoning social media industry (Knoll, 2016). By 2020, social media advertising revenue in USA alone will climb over 30 billion dollars (Meola, 2016).

Despite these lucrative advantages of social media advertising, more than one-third of users consider online community advertising is annoying (Nielsen, 2012). A recent survey reveals that around half of browsers in developed economics consider that they are more intrusive now than 2–3 years ago (Hubspot, 2016). A number of failure cases (e.g., MySpace and Apple’s Ping) have occurred due to user–advertiser conflicts. Indeed, user–advertiser conflicts impede users’ participating intention into online communities, which has a large negative influence on user’s perception of community advertising (Taylor et al., 2011).

Substantial research efforts have investigated the acceptance of online community advertising from users’ perspective (e.g., Chi, 2011; Taylor et al., 2011) that determines the survival of the community (Schumann et al., 2014). Another research angle concerns effects of a pleasant online community (Knoll, 2016), from the perspective of users’ self-motivated communications (Wang et al., 2015). Recent advertising researches (e.g., Bang and Lee, 2016; David, 2016; Bidmon, 2017) found that the proper usage of interpersonal intimacy and attachment can stimulate consumers’ positive reaction to advertising. As a matter of fact, communication is users’ original intention to participate an online community. As an example on the other side of the coin, Reddit initiated its first advertising test in 2016 (Reddit, 2016), which is a popular American social news aggregation and discussion platform. It turned out that the majority of users demonstrated their understanding and willingness to continuously support the community, although some opponents existed. Practically, in the Reddit case, user’s positive perception of community advertising can be due to several distinct mechanisms Reddit created to maintain cohesive communities among users, such as programs (e.g., “Ask Me Anything”), community’s etiquette (e.g., “redditors do not want to advertise for you, they want to talk to you”), and the censorship policy (e.g., “we will not ban legal content even if we find it odious”). Therefore, it’s critical for an online community to study users’ perception and acceptance to social media advertising from the viewpoint of communication mechanisms.

This work follows this direction to explore how online community-supported social communications affect users’ perception and responses toward advertising. We contend that one effective path to gain users’ positive perception of advertising could be communication mechanisms. That is, by employing an elaborated communication system, online community can facilitate the intra-community communications and then users’ positive reaction to advertising (Yang et al., 2017a). However, little is known about how communication mechanism influence users’ advertising reaction and what factors play the critical roles.

This work developed a conceptual model based on the social bond theory (SBT), encapsulating 9 constructs and 10 hypotheses extracted from the extant online community advertising literature. In this study, following Knoll (2016), online community advertising is identified as “persuasive and planned communication by advertising professionals deliberately placed on third-party websites”. We collected data by a snowballing sampling approach from 327 validated participants to examine our model and identified hypotheses. This research yields several important findings that provide critical managerial insights for both online communities and advertisers. First, social communications can effectively expedite users’ behaviors to be in accordance with a community’s interests. Second, among four prevalent aspects of social communications, except for recognition for contribution, the other three have high (or at least medium) influence level and could produce positive response to social media advertising. Third, social bonds can raise acceptance and responses toward community advertising, with users’ group intention as an intervening factor.

The rest of this paper is organized as follows. Section “Theoretical Background” presents a brief literature review related to SBT and social media advertising. Then, our theoretical framework and related hypotheses are developed in Section “Theoretical Framework and Hypotheses”. Section “Research Methodology and Results” reports the research methodology and report statistical results. Finally we discuss main research findings, empirical suggestions, and outline future research perspectives in Section “Conclusion and Discussion”.

## Theoretical Background

Online community is defined as “an aggregation of individuals or business partners who interact based on a shared interest, where the interaction is at least partially supported or mediated by technology and guided by certain protocols and norms” in Porter and Donthu’s (2008, p115) work. An online community not only provides an open discussion space, but also facilitates the building of social orders (such as social norms, social interactions, and bonds), and entitles users to take on various statuses in order to maintain the systemic equilibrium (Tsai and Bagozzi, 2014).

Actually, the core of an online community includes the shared rituals and traditions, the sense of moral responsibility, and the obligation to the community (Muniz and O’Guinn, 2001). Thus, social behavior theories are widely applied to study interactions between users and online communities (e.g., Dholakia et al., 2004; Porter and Donthu, 2008; Oliveira et al., 2016). Among these theories, SBT focuses on the socialization theme, i.e., why an individual attaches to the society (Hirschi, 1969)? People regulate their behavior in accordance with social norms when strong social bonds existed between themselves and their target group. SBT can explain how to maintain adults’ conventional behavior not only within the offline organization environment (Cheng et al., 2013), but also the social networking website setting (Marcum and Higgins, 2014). The more the conventional interaction conducted by online community users, the more close the interpersonal relationship can be achieved (Ren et al., 2012). Meanwhile, one of the major successful reasons for an online community is bond-based attachment, the mechanism of which is frequently explained by SBT (e.g. Lim, 2013; Shih and Huang, 2014). Users attach to other members and conduct altruist behaviors when the social bonds are constructed (Ren et al., 2012). Based on these characteristics, SBT is applicable to explain the influence of social communications on users’ advertising attitude.

Hirschi (1969, p.16) argued that in order to generate and maintain social bonds to the target groups, an individual should have “*attachment* to families, *commitment* to social norms and institutions, *involvement* in activities, and the *belief* that these things are important.” As for an individual internalized by these four elements, she will adapt her behaviors in accordance with conventional guidelines approved by the target social group (Tischler, 2013). Additionally, when an individual attaches closely to other members in a society, she is prone to believe in common moral values of the society. *Attachment* refers to affective ties or respect that an individual forms toward significant others in a social group (Hill and Pollock, 2015). In addition, peer relationship is an alternative dimension of attachment (Hirschi, 1969). In online community setting, users who are attached to other members (e.g., senior members and close friends) will essentially be motivated to display behaviors that are accepted by the society. *Commitment* is related to an ambitious goal that an individual is eager to achieve, e.g., the high-level education and high-status reputation (Hirschi, 1969). *Commitment* is considered as a rational cost analysis of conducting certain activities that are accepted or rejected by the frame of reference (Chriss, 2007). In online community context, committed users are inclined to invest tremendous time and effort to seek certain goals or end status that can match their aspiration. *Involvement* refers to an individual’s actual engagement in conventional activities that can lead to the anticipated success and status (Cheng et al., 2013). The more socially approved activities an individual is engaged, the more positive feedback is obtained from his peers. In online community background, the conventional activities are tasks that breeding the healthy growth of the very online community. *Belief* refers to the degree to which an individual accepts the moral element of the central social value system (Hirschi, 1969). *Belief* exerts the indirect control, rather than the coercive power, on an individual’s behaviors. Therefore, an individual identifies himself as a member of conventional society will care about others’ perception toward himself and internalizes the expectations of others of the targeted group (Chriss, 2007). In online community situation, belief equals to the value system shared by all members as advocated by Muniz and O’Guinn (2001).

Social bonds are verified and can generate outcomes benefiting online community, e.g. contribution, altruism, and improved quality of online community (e.g., Ren et al., 2012; Shih and Huang, 2014; Chung et al., 2016). In this sense, users would cooperate with community platform in a proactive manner in order to integrate into the target online community. Online community advertising could benefit from users’ aggregations and engagement. However, there are ubiquitous critics to online community advertising, probably due to the negative user reactions (Schumann et al., 2014). We argue that, the critical reason reside in the fact that current online community advertising services focus on utilitarian rather emotional strategies (Zhu et al., 2012; Niven et al., 2015). Once users engaged with an online community, they incorporate themselves into the community and contribute their own resource and competences for the community’s benefits (Verhagen et al., 2015). We contend that social bonds build up the intimacy among users and then leads users to aware the importance of advertising toward community’s survival.

Extant research identifies the correlation between members’ interaction and social bonds. For instance, a positive relationship between social bonds and adult school members is supported in online learning literature (Han and Johnson, 2012). Moreover, social interaction is also found positively related to social bonds among various online communities (Fiedler and Sarstedt, 2014). In other words, the more the interaction among users, the more concentrated the contacts became, the more evident the bond can be perceived by users (Lim, 2013). On the other hand, online communities apply various computer-mediated communication technologies to encourage users’ interactions (Porter and Donthu, 2008). The resulting functional and social benefits (Dholakia et al., 2009) generate users’ reciprocal and cooperative behaviors (Ortiz-Cordova et al., 2015), which drives users to perform community-supporting behaviors (Chan et al., 2014; Wang et al., 2016). Therefore, we contend that social communications can positively affect users’ sense of social bonds and sequential reaction to online community advertising. As far as we knew, none of prior studies examined the influence of social communications on consumers’ perception on advertising. This research makes an attempt to analyze the influence of social communications on users’ engagement with community and their subsequent intention to accept advertising, and actual response toward advertising.

## Theoretical Framework and Hypotheses

As a number of research employ social bonds-based attachment to examine various online community phenomena (e.g., Ren et al., 2012; Shih and Huang, 2014; Chung et al., 2016), we adopt their descriptions to delineate the social bonds in this study. Social bonds represent the degree of mutual friendship and liking shared by relationship partners (Wilson, 1995), or the norms and standards of conduct that are required for an ongoing relationship (Dwyer et al., 1987). Therefore, in this study, social bonds are defined as the degree of emotional and social ties link between individuals within online communities, which indicates the degree of closeness between an individual and online communities (Ren et al., 2007).

Online community applies various communication approaches to fulfill users’ informational and social interaction demands. Computer-mediated communication creates relationship affinity, which facilitates the mutual respect, mutual commitment, and equal status among users (Ou et al., 2014). In addition, the harmonious interpersonal relationship is helpful for the maintenance of an online community (Settanni and Marengo, 2015). The role of computer-mediated communications can be understood as an antecedent of close online relationship and commitment to the community (Zhou et al., 2016). Social communications essentially provide the common media channel for users’ joint action, enabling them to experience positive feedback and social information. Such cooperative activity provokes elaborate processing of shared object, a sense of social status categorization, strong intentional stance toward shared goal, and feeling of common success (Wolf et al., 2015). These consequences then will lead to social bonds among community users. In short, social interaction provides opportunities for users to develop cohesiveness, shared value, and sense of collaborative relationship (Fiedler and Sarstedt, 2014), and is considered by researchers as a cause for social bonds within online communities (Ren et al., 2007). We contend that communication mechanisms offered by online communities establish the social interaction opportunity for users.

Online communication tools not only transfer community value to users, but also enhance the intimacy between users (Yang et al., 2017a). Practically, online communities adopt various measures to inspire active information sharing and high-quality content generation. The widely used approaches can be categorized into four types, namely: support for communication, perceived community value, recognition for contribution, and freedom of expression (Kang et al., 2007; Kim et al., 2008; Chan et al., 2014).

Support for communication describes the extent to which the approaches, assistance, and opportunity provided by an online community with the purpose of stimulating users’ communication (Kang et al., 2007). A cozy atmosphere or harmonious environment is a fertile ground for interaction and relationship development. Online community frequently host single theme activity to increase intimacy among users. For instance, Reddit’s Ask Me Anything invites users to interact with guest and attracts tens of thousands of users’ participation. A user can communicate with higher community status users or peer users with such opportunity. The intimacy between a user and those significant users can receive enough growing space, which is similar to the attachment assertion of SBT. In addition, the more joint actions conducted by users, the stronger the social relationship among user, the bigger the opportunity that a social bonds can emerge (Wolf et al., 2015). From this perspective, support for communication can be understood as playing the function social interaction. We thus posit:

H1: support for communication has a positive effect on social bonds.

Perceived community value is illustrated as the degree to which users certain the needs and benefits they pursue are in accordance with the online community’s declared value system (Kang et al., 2007). For instance, Reddit’s users hold the common belief that the community is an open cyberspace belonging to themselves. The shared value of online community serves as the framework of reference, incorporating users’ relation to the environment, and to the social system (Lu and Yang, 2011). The function of perceived community value thus equals to pattern-maintenance system, as belief in SBT. Further, the core value system of online communities solidify the relationship and strengthen group cohesion among users (Muniz and O’Guinn, 2001). Individual user would evaluate the match between his demands and what the community requires. Once the assessment accomplished, a user applies community values and norms as his code of conduct, leading him to be embedded into the bond-based relationship with other users. We thus postulate:

H2: perceived community values have a positive effect on social bonds.

The interactive communication among users is mainly achieved in terms of various user-generated content (UGC). Recognition for contribution is described as the extent to which an online community approves UGC or other altruistic behaviors, in the manifestation of monetary or psychological rewards (Kim et al., 2008). Recent research suggested a user’s psychological well-being within an online community is related to his UGC (Rae and Lonborg, 2015). For instance, high-quality content generators in Reddit are awarded with gold “creddits”, which represents a kind of honor for users. Such kinds of recognition increase user’s reputation and status among other community peers. SBT argues that high-level social goals, such as reputation and personal achievements, are worthy of the spending of an individual’s time and energy (Chriss, 2007). Extant literatures argue that the reputation and personal achievement are purposes of information contribution in online community context (Dholakia et al., 2009; Nov et al., 2010). It appears that these results overlap a bit the commitment argument in SBT. On the other side, the UGC process normally coupled with the revelation of self-disclosure information (Teichmann et al., 2015), which is found positively associated with social bonds in online communities (e.g., Lim, 2013; Cress et al., 2014). It can be interpreted as that the contribution made by committed user resulting in the presentation of personal information and which further solidifies the social bonds among users. We, therefore, hypothesize:

H3: recognition for contribution has a positive effect on social bonds.

Anonymity mechanism of online community allows more convenience, less moral pressure, and more opportunities for users to give out their own opinions (Hercheui, 2011). A temptation to potential online community participants is that they are free to express diverse viewpoints (Yang et al., 2017a). Freedom of expression in cyberspace refers to the extent of which an online community’s infrastructure can facilitate and protect users’ rights to express diverse viewpoints (Kim et al., 2008). When policies restrict users to express their opinions, they will commit deviant behaviors. SBT argues that the more time an individual spend in high-quality social activities, the shorter the distance between him and target group is perceived by himself, the less likely he will engage in deviant behavior (Hill and Pollock, 2015). A speech censorship which tolerating various opinions motivates users to participate in high-quality social interaction will increase users’ willingness to build friendship with other users, such as Reddit’s censorship policy of “we will not ban legal content even if we find it odious”. In fact, the more online interaction an online community user involved in, the more social investment he will make, and the stronger the emotional and conventional bonds between him and other users (Yohannis and Sastramihardja, 2009; Zhu and Ratner, 2015). Therefore, getting users actively involved into high quality free view expression and interpersonal communication are supposed to be effective in generating close social relationship. We, therefore, posit:

H4: freedom of expression has a positive effect on social bonds.

Online community users’ group intention is initially defined as their commitment, and agreement to participate a joint action, based on the premise that members consider themselves as part of the online community (Bagozzi and Dholakia, 2002). As online community’s work is to educate users to understand the importance of advertising and accept it, we conceptualized group intention as community members’ acceptance and evaluation to advertising in this study. Existing literature suggests group intention arose from a user’s need for approval from online community’s significant peers (Bagozzi and Dholakia, 2002). The underlying mechanism of such process is similar to the theme of social bonds. Several research found that online community users who are tied with other users by social bonds perceiving their community as more cohesive and willing to remain in this community (Ren et al., 2007). In addition, online community users incline to generate a “habit of cooperation” and perform collective activity when they have regular interaction with other users (Zhao et al., 2016). For that matter, the more intense the social bonds being felt and held by users, the higher the likelihood that they will form a group intention. We thus hypothesize:

H5: social bonds have a positive effect on group intention.

Advertising relevance refers to certain elements of advertising are considered as meaningful, useful, and valuable to community users in this study (Smith et al., 2007). Previous literature suggests that, within the context that people are less care functional demands, topicality of ad message is the most influential factor in determining people’s relevance evaluation (Xu and Chen, 2006; Xu, 2007). In this vein, users might accept advertising if ad topic match the theme of online community. In addition, such relevance evaluation is more related to behavior rather the message contend (Xu, 2007). Such that, when community members possess positive group intention of accepting advertising, they are incline to support this group activity due to they believe that advertising is in accordance to the community’s maintenance. As a result, community users should shape an impression that advertising is relevant to the community and themselves. We hence, postulated that:

H6: group intention has a positive effect on ad relevance.

Perceived advertising value refers to user’ subjective evaluation related to advertising’s relative worth or utility in total (Ducoffe, 1995). Extant literature argued that user’s perceived value reflects the social dimension of their consumption behaviors (Gallarza and Saura, 2006). Under the social interaction context, the perceived value emerges via adoption or acceptance of an object that shared with others (Williams and Soutar, 2009). Social relationship and related experience that valued by online community users underscore the social aspect of perceived value. When community users possess a group intention to agree the existence of advertising, they are likely to perceive the social respect of perceived value from accepting or consuming the advertising. As a result, while community users all agree to accept online advertising, the perceived value from common adoption of advertising increases as well. We thus postulated:

H7: group intention has a positive effect on ad value.

Information relevance studies depict a positive relationship between consumers’ perceived relevance and value of the document at hand (Xu and Chen, 2006). Advertising literature similarly indicated that consumers’ cognitive assessment determines how they assess advertising value (Ducoffe, 1996). We contend that when users perceive advertising relevant to the theme of community, they should realize the usefulness of such advertising. In addition, perceived value is commonly understood as the trade-off between consumers’ assessment of benefits and sacrifices (Zeithaml, 1988). The more advertising information relevant to users, the lesser the effort and time are paid by users, the higher the utility from such advertising message can be gained by users. As a result, user’s perceived ratio between ‘get’ and ‘give’ subsequently increase the perceived value of this advertising. We posit that:

H8: Ad relevance has a positive effect on ad value.

Users’ response to online advertising captures a series of behavioral responses toward advertising, including notice, attention, ad-click, and purchasing products (Rodgers and Thorson, 2000). Consumers perceive the ad relevance when ad message elements are meaningful and reflect their subjective experience (Smith et al., 2007). In addition, perceived relevance facilitates consumers to engage with an ad within online advertising context (Spielmann and Richard, 2013). Positive outcomes (e.g., notice, favorable attitude, and purchase intention) can result from an ad when which is considered in line with the theme of online community by users. Popular viral video advertising (i.e., “Will it Blend?”) is the example of employment of advertising message or content relevance. Thus, the more relevant the advertising perceived by users, the more usefulness of advertising felt by users, the higher the possibility of positive reaction to advertising. We hypothesize:

H9: Ad relevance has a positive effect on users’ ad response.

Advertising value is an important antecedent of consumer attitude (Ducoffe, 1996). Attitude theories indicate that consumer’s positive attitude influence his behavior. Moreover, perceived value studies found that perceived value improves outcomes of consumer’s attitudinal and behavioral reaction to service or products (Gallarza and Saura, 2006). That is, the higher the value of advertising perceived by community user, the positive his attitude toward this advertising, and consequently the positive his response toward advertising. In addition, online advertising research related to the relationship between perceived advertising value and consumers’ advertising response verified the existence of such association (e.g., Taylor et al., 2011; Kelty et al., 2012). As a result, we postulate:

H10: Advertising value has a positive effect on users’ ad response.

## Research Methodology and Results

### Sampling and Data Collection

Participants of this survey were recruited through a web-based survey. We used the pay service of a leading Chinese online survey company. This company reward members with several bonus points, which can be used to exchange gifts, after their finished questionnaires are qualified. We used snowballing sampling approach since which is effective in locating actual users. The selection criterion is a user must hold at least 3 months registered membership of his favorite online community and, at least, invested at least 4 h a week in visiting that website. We initially invited some random users within several big online communities and ask them to invite more online community users. With the beginning of formal questions, a scenario requested participants to provide a list of their most visiting online communities and image that “he is right now conducting regular activities in his favorite online community, such as browsing, chatting, and posting threads.”

The consent form informed was presented to participants at the beginning of questionnaire, including the research purpose, a guarantee of the anonymity of their identity and the security of their personal information, and the assurance that participants could complete or withdraw at any time. Participants were told that all responses provided by them were only for academic purposes. Full review and approval by an ethics committee was not required for this study in accordance with the institutional and national requirements.

A total of 327 qualified participants were recruited. In general, the average time is 1.56 years for membership duration and is 6.2 h/week for website visiting time. Gender of participants is roughly distributed (male = 51.2%, female = 48.8%). The range of participants’ age is 20–31 years old (mean = 26.7 years), among which 90% were 20–29 years old. The most mentioned websites are QQ zone and Tianya Club, all of which are popular online communities.

### Measures

All scales were adopted from existing literature. The reliability and validity of these measurements are testified by a number of empirical studies. Necessary semantic edition of items was conducted to adapt to research context. Questionnaire was then processed by means of translation and back-translation techniques, ensuring participants can fully understand items’ original meaning. A summary of constructs and related items are demonstrated in **Table 1**.

**Table 1:** Measurements, items, and construct loading.

Construct	Items	Factor Loading
Support for communication (SC) Composite reliability (CR) = 0.92, α = 0.91, average variance exacted (AVE) = 0.73	The community provides an effective bulletin board where participants communicate.	0.81
	The community provides various means to support member communication such as chatting room, e-mail service, member search service, game, etc.	0.79
	The community supports various events for members to experience together.	0.92
	The community provides various supports for members to get together.	0.90
Perceived community values (PCV) CR = 0.91, α = 0.91, AVE = 0.76	The community provides a clear purpose of the community	0.8
	The design of the community site makes it easy for participants to know for what purpose the community exists	0.91
	The community provides a clear message about for whom the community exists	0.9
Recognition for contribution (RC) CR = 0.89, α = 0.89, AVE = 0.73	The community provides proper rewards to active members for their efforts	0.89
	The community provides strong supports for various active member activities	0.86
	The community shows proper gratitude to actively participating members	0.81
Freedom of expression (FE) CR = 0.83, α = 0.73, AVE = 0.72	The community proactively embraces negative discussions or opinions about the brand from members	0.84
	The community positively deals with complaints about the brands or other services from members.	0.85
Social bonds (SB) CR = 0.92, α = 0.92, AVE = 0.61	The community friendship and relationship is meaningful to me.	0.79
	I feel bad if my concerned friend leave this community.	0.76
	I am similar to members in some respects of this community.	0.73
	I will recall some friends and activities that we all participated in when this community is mentioned.	0.82
	I will try my best if I make promise to community friends.	0.82
	I will spend my time to communicate with community members.	0.78
	I tend to trust community friends to some extent.	0.76
Group intention (GI) CR = 0.92, α = 0.92, AVE = 0.75	Members of this community consider community advertising to be normal.	0.87
	Members of this community consider community advertising a part of the community offering.	0.97
	Members of this community consider community advertising a source of information.	0.81
	Members of this community tend to accept community advertising.	0.81
Perceived Ad relevance (ARL) CR = 0.92, α = 0.93, AVE = 0.67	Advertising in this community is relevant to me.	0.67
	Advertising in this community is important to me.	^a^
	Advertising in this community means a lot to me.	0.74
	Advertising content in this community is matching the community.	0.89
	Advertising in this community is pertinent to the community.	0.84
	Advertising in this community is relevant to the theme of this community.	0.93
	Advertising in this community is in accordance with community content.	0.83
Perceived Ad values (AV) CR = 0.95, α = 0.96, AVE = 0.81	The community advertising to some extent is useful for me.	0.96
	The community advertising to some extent is valuable for me.	0.96
	The community advertising to some extent is important for me.	0.92
	The community advertising is a convenient source of product information.	0.83
	The community advertising is a good source of product information.	^a^
	The community advertising supplies relevant product information to my interest.	0.82
Ad response (ARP) CR = 0.86, α = 0.86, AVE = 0.5	I will pay attention to advertisements shown in this community.	0.72
	I will have the impulse shopping intention when I see some advertisements.	0.67
	I will click advertisements that I am interested in.	0.73
	I will glad to have a look on advertisements if they are relevant to me.	0.70
	I will search for related information about advertisements that I am interested in.	^a^
	I have searched for the product company website about advertisement that I am interested in.	0.75
	I have purchased the products showed by advertisements.	0.67

*^a^Item was deleted due to low factor loading.*

Four aspects of social communications namely support for communication, perceived community values, recognition for contribution, and freedom of expression were selected from extant similar studies (e.g., Kang et al., 2007; Kim et al., 2008). A total of 13 items were adopted by this study. The measures of social bonds were based on Hirschi’s (1969) seminal work and following studies (Cheng et al., 2013), including seven items. Scales of group intention were chosen from Bagozzi and Dholakia (2002) and Dholakia et al.’s (2004) research. This construct was aimed to examine the extent to which users’ acceptance of online advertising. The scales of perceived advertising value were picked out from Ducoffe’s (1995, 1996) advertising value model. The measures of perceived advertising relevance were based on Laczniak and Muehling’s (1993) research. The measures of response to online advertising were adopted from Rodgers and Thorson’s (2000) study. In addition to several related generic advertising metrics (i.e., attention, memory, attitude, and purchase), this construct also includes some response measures (i.e., exploratory behaviors) specified for online community advertising. All measurement items were scored on seven-point Liker scale, ranging from “strong disagree (=1)” to “strongly agree (=7)”.

### Results

We employed structural equation modeling (SEM) to examine the hypotheses. We follow a two-stage procedure: verification of reliability and validity of the measurement model, followed by examination of structural model and hypotheses (Hulland, 1999; Rönkkö et al., 2015). We assessed the reliability and validity through confirmatory factor analysis (CFA) using AMOS 22.

### Measurement Model Assessment

Several index requirements should be met for a good model fit, χ^2^/*df* should less than 3.0 (Kline, 2010), the goodness-of-fit (GFI) should be close to 0.9 (Doloi et al., 2010), the normed fit index (NFI) should be large than 0.9 (Kline, 2010), the comparative fit index (CFI) should be large than 0.9 (Kline, 2010), and the root mean square error of approximation (RMSEA) should be less than 0.08 (Browne and Cudeck, 1993). To ensure acceptable estimate-to-observation ratios, we divided all measures into theoretical related constructs (Bentler and Chou, 1987). Support for communication, perceived community value, contribution for recognition, and freedom of expression are included in model 1. Group intention, perceived ad relevance, perceived ad value, and ad response are included in model 2. These two measurement models were examined in terms of reliability, convergent validity, and discriminant validity. The results of CFA for both models depicted the acceptable fit (Model 1: χ^2^ = 330.06, *df* = 133, χ^2^/*df* = 2.48, GFI = 0.9, CFI = 0.96, NFI = 0.93, and RMSEA = 0.07; Model 2: χ^2^ = 389.95, *df* = 163, χ^2^/*df* = 2.39, GFI = 0.89, CFI = 0.96, NFI = 0.94, and RMSEA = 0.07).

Cronbach’s α is used to test reliability of constructs (Rönkkö et al., 2016). Results in **Table 1** show that α-values for all constructs exceed the suggested value of 0.7. Convergent validity is acceptable if construct item loading exceed 0.6, the composite reliability (CR) and the Cronbach’s α larger than 0.7, and the average variance exacted (AVE) above 0.5.

**Table 1** showed the results of α value, CR, AVE, and factor loading. The values of α and CR for all constructs were greater than 0.7. The smallest AVE value was of advertising response (0.5), reaching the threshold value of AVE. After deleting disqualified items, the smallest factor loading value of remaining item was with regard to advertising response (0.67). In addition, no significant cross-loading was detected. Results of item loading, α-value and CR, and AVE depicted the acceptance of convergent validity. As for discriminant validity, we followed the suggestion that the between-construct correlation should be lower than the square root of AVE of each construct (Fornell and Larcker, 1981).

**Table 2** demonstrated the square root of AVE were greater than correlation values under the off-diagonal. Thus, discriminant validity was confirmed. Next, we examine common method bias due to self-reporting survey. Harman single factor test was performed in here. Specifically, all constructs are examined by exploratory factor analysis, and common method bias is eliminated if on one factor variance explained more than 50% constructs (Liang et al., 2007). Eight factors with eigenvalues greater than one were extracted, and the first factor explained 25.29% variance before rotation. Hence, common method bias was not a significant problem.

**Table 2:** Correlation matrix, square root of AVE, and descriptive statistics.

	1	2	3	4	5	6	7	8	9
SC	**0.86**								
PCV	0.644^∗∗^	**0.87**							
RC	0.640^∗∗^	0.629^∗∗^	**0.85**						
FE	0.599^∗∗^	0.555^∗∗^	0.614^∗∗^	**0.85**					
SB	0.538^∗∗^	0.482^∗∗^	0.447^∗∗^	0.477^∗∗^	**0.78**				
GI	0.375^∗∗^	0.428^∗∗^	0.372^∗∗^	0.328^∗∗^	0.356^∗∗^	**0.87**			
ARL	0.209^∗∗^	0.332^∗∗^	0.304^∗∗^	0.259^∗∗^	0.212^∗∗^	0.213^∗∗^	**0.82**		
AV	0.305^∗∗^	0.399^∗∗^	0.329^∗∗^	0.337^∗∗^	0.396^∗∗^	0.632^∗∗^	0.426^∗∗^	**0.90**	
ARP	0.270^∗∗^	0.355^∗∗^	0.383^∗∗^	0.343^∗∗^	0.317^∗∗^	0.492^∗∗^	0.751^∗∗^	0.776^∗∗^	**0.71**

Mean	4.62	4.70	4.42	4.15	4.34	4.30	3.80	3.76	3.73
*SD*	1.50	1.56	1.50	1.40	1.39	1.54	1.13	1.61	1.24

*Diagonal elements (bold) are the square root of AVEs. Off-diagonal elements are the correlations among constructs. ^∗∗^*p* < 0.01.*

### Structural Model and Hypotheses Testing

**FIGURE 1:**
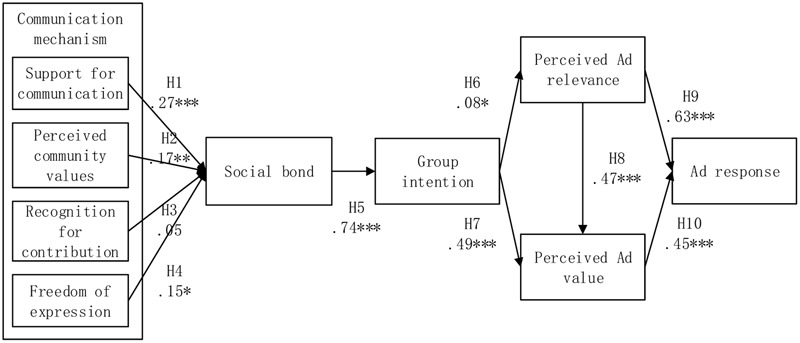
Structural model and analysis results. ^∗^*p* < 0.05; ^∗∗^*p* < 0.01; ^∗∗∗^*p* < 0.001.

We performed the structural model analysis to examine hypotheses via maximum-likelihood estimation after the verification of measurement models. The research model in **Figure 1** illustrated the relationships among nine constructs, each of which was represented by a single indicator using summated scales (Price et al., 1995). The fit indices indicated a good fit for the research model using the same criteria for measurement model (χ^2^ = 50.61, *df* = 18, χ^2^/*df* = 2.81, GFI = 0.97, CFI = 0.98, NFI = 0.97, and RMSEA = 0.08). The squared multiple correlations (SMC, *R*^2^) for the structural model for social bonds (*R*^2^ = 36.3%) and group intention (*R*^2^ = 32.3%) were relatively high.

We postulated that social communications have a positive influence on the social closeness via H1–H4. Results in **Table 3** indicated the positive relationships between social bonds and support for communication (γ = 0.27, and *p* < 0.001), perceived community values (γ = 0.17 and *p* < 0.05), and freedom of expression (γ = 0.15, *p* < 0.05), respectively. Hence, H1, H2, and H4 are supported. Besides, the relationship between recognition for contribution and social bonds was positively insignificant (γ = 0.05, and *p* > 0.05). H3 was not supported. Social bonds were found to has positive effect on group intention (γ = 0.74 and *p* < 0.001), confirming H5. Next, H6, H7, and H8 addressed the relationship among group intention, perceived ad relevance, and perceived ad value. Group intention was identified to positively influence perceived ad relevance (β = 0.03 and *p* < 0.05) and perceived ad value (β = 0.49 and *p* < 0.001), supporting H6 and H7. Likewise, the results in **Table 3** depicted perceived ad relevance was positively associated with perceived ad value (β = 0.47 and *p* < 0.001), supporting H8. Finally, H9 and H10 addressed the relationship between ad response and perceived ad relevance and perceived ad value. The relationships between advertising related variables are as well posited as positively connected. Both perceived ad relevance (β = 0.63 and *p* < 0.001) and perceived ad value (β = 0.45 and *p* < 0.001) were found to positively connected ad response. Thus, H9 and H10 were confirmed.

**Table 3:** Path coefficient and *p*-value.

		Path coefficient	*p*-value
H1	SC → SB	0.27***	<0.001
H2	PCV → SB	0.17**	0.01
H3	RC → SB	0.05	0.42
H4	FE → SB	0.15*	0.02
H5	SB → GI	0.74***	<0.001
H6	GI → ARL	0.08*	0.03
H7	GI → AV	0.49***	<0.001
H8	ARL → AV	0.47***	<0.001
H9	AR → ARP	0.63***	<0.001
H10	AV → ARP	0.45***	<0.001

*^∗^*p* < 0.05; ^∗∗^*p* < 0.01; ^∗∗∗^*p* < 0.001.*

To sum up, most of the hypotheses were supported. Our research model illustrates the connection roadmap of how social communications affect users’ perception and response to online community advertising. Specifically, communication techniques aimed at establishing positively association between the sense of attachment, belief, and involvement and social bonds among all users. Community users were then consolidated into an entity that holding similar emotional intention. The consensus of accepting advertising as a means to sustain the growth of community facilitated the internalization of positive perception toward advertising. Lastly, perceived ad relevance and perceived ad value were both found to positively relate to users’ ad response.

## Conclusion and Discussion

### Summary of Results

This study explored influence of social communications on users’ reaction to social media advertising in an online community. Our research yields several critical findings.

First, social communications have a normative power to build social bonds among users and users’ attachment and emotional belongingness with an online community, by enhancing the interactions among users in an online community. That is, according to SBT, users adapt their behaviors to an online community when they are willing to connect to it (Chung et al., 2016; Zhou et al., 2016). Previous literature (e.g., Ren et al., 2012; Fiedler and Sarstedt, 2014; Chung et al., 2016) identified the positive yet limited influence of social bonds on the group intention in a community. Nonetheless, our finding indicated that social bonds can exert the significant influence.

Second, four communication aspects have different degrees of influence on social bonds. Specifically, *support for communication* illustrates the strongest influence on social bonds in an online community, followed by *perceived community value*, and *freedom of expression* has the least, while the effect of *recognition for contribution* is not statistically significant. This phenomenon can be explained as follows. The finding about *support for communication*, which offers users opportunities to contact their target users of interest, is consistent with previous observations (e.g., Kang et al., 2007): communication involvement might be users’ base intention to participate and attach to a community. As for *perceived community value*, users take it as a common belief to adjust their behaviors, although the computer-mediated communication might dilute users’ perceived community value (Wellman et al., 2001; Lee et al., 2014). This might explain the effect of *perceived community value*. As for *freedom of expression*, it appears to have two-folded effects. That is, a tolerant environment not only entitles users to express diverse opinions that can promote their involvement in social interactions, but also provide opportunities for spam messages. Thus, users have to bear a moderate level of censorship. A possible explanation for the insignificant relationship between *recognition for contribution* and *social bonds* reside in users’ contribution motivation. Actually, active information sharers are normally entertainment seekers (Alarcón-del-Amo et al., 2015). That is, in our case, entertainment might be a major motivation under users’ online behaviors (Lee and Ma, 2012). Overall, our findings are consistent to previous research (e.g., Kang et al., 2007; Kim et al., 2008) that *support for communication* had the strongest influence, and the sequence of remaining three aspects in terms of influence magnitudes is *perceived community value, freedom of expression*, and *recognition for contribution*.

Third, social bonds can raise users’ acceptance and responses toward community advertising, with group intention as an intervening factor. Moreover, the transition effect from users’ group intention to advertising response is specified by *perceived ad relevance and value*. Compared to *perceived ad relevance*, group intention is more related to *perceived ad value*. This might be because the nature of group intention makes users pay more attention to overlapped value (Bagozzi and Dholakia, 2002). Furthermore, the association between users’ response toward advertising and *perceived ad relevance* is stronger, compared to *perceived ad value.* Perceived ad relevance in online settings dependents on the context where users are located (Xu and Chen, 2006). That is, a bonded online community constructs an edge-cutting information environment for users (Cress et al., 2014), thus demands a high relevance from advertised products. Thus, users are more inclined to react positively to advertisements with a great amount of relevant information with respect to the context.

### Theoretical Implications

This study makes several contributions to the extant social media advertising literature. First, we apply the SBT to investigate how social communications affect users’ responses to advertising. Existing studies applied the attachment-based principle to examine online community’s influences on users’ attitudes and behaviors (e.g., Chung et al., 2016; Oliveira et al., 2016), found that bond-based attachment exerts limited influence (Ren et al., 2012; Fiedler and Sarstedt, 2014). This work takes a different perspective, and our findings demonstrate that influence of social bonds can be enhanced by proper inducement mechanisms (i.e., social communications). Second, we enrich studies related to the performance of communication approaches in an online community, by revealing the different levels of influence exerted by four communication aspects. Third, the research outcome complements the literature regarding the range of factors in affecting consumers’ response toward online advertising. Existing research found social bonds can increase users’ attitudes toward advertising (e.g., David, 2016; Bidmon, 2017). Our study further identified that such influence can be transmitted in terms of group intention, i.e., the antecedent of users’ perception (i.e., *perceived ad relevance* and *perceived ad value*) and response toward advertising.

### Managerial Implications

We believe that there exists a tradeoff between the fulfillment of users’ social appeals and the advertising demands of online communities. This study provides critical managerial insights to managers of online communities and advertisers in the field of social media advertising. First, our research outcome can help managers of online communities understand the role of social bonds in the ecosystem of an online community. An online community can influence users’ behaviors through proper techniques on the close attachment. To achieve this purpose, online community should continuously develop new and interesting communication tools to strengthen users’ stickiness. The top design principle should be creation of rich communication environments, e.g., offering rich choices of communication forms and flexible multimedia channels, to fulfill users’ interaction needs. The second principle is to highlight community value, build norms among liked-mind, and influential users to improve users’ perception of community’s capital that congruent with its value declaration. A third strategy for community managers is to allow diverse opinions and strengthening this impression to users.

Second, our findings remind stakeholders that users’ group intention is capable of boosting users’ responses toward social media advertising. Community managers can conduct some propaganda campaigns to persuade users to understand community’s long-term interest. Moreover, community manager can use group intention as an advertising strategy adjustment metric. In addition, advertisers could cooperate with online community to periodically need to track and assess users’ group intention by analyzing their behaviors. The assessment results could help decision makings on ad displacement. Advertisers could adapt their advertising message (e.g., ad slogans and propaganda words) to deliver messages allying with the online community.

Third, advertisers should improve the perceived advertising value and relevance to obtain better users’ response toward social media advertising. Advertisers should design ads highlighting expected values (e.g., entertainment value, functional value, and social value) for potential consumers, and create word-of-mouth (WOM) effects (Choi and Lee, 2015). In the meanwhile, ads should be designed and delivered aligning with themes of an online community to enhance the “best match” (e.g., ad relevance) between a specific user in a specific context and a suitable advertisement (Yang et al., 2017b).

### Limitation and Future Study

The limitations of this study are related to data collection and types of websites and advertisements. First, as for data collection was conducted in a single country and participators were primarily students. People’s reaction to social bonds in different cultures varies (Krohn et al., 1984). Second, the age of respondents in our survey ranges from 20 to 31. It might have two reasons. According to CNNIC (2016), young adults (18–29 years) occupy the highest percentage among all social networking website users. Moreover, adults (older than 30 years) may not be interested in participating online survey. Thus, we need to be cautious to apply our findings users with other ages. Future study will attempt to obtain a representative sample of online community users by employing effective sampling techniques. Third, the most visited websites under this study are classified as social networking and company-supported. As different online communities serve different purposes, the valence of users’ reactions to advertising might vary across different websites types of and users’ demands. The findings from extant similar studies (e.g., Kang et al., 2007; Chan et al., 2014; Yang et al., 2017a) implies that websites characteristics might cause different user reactions to social communications. Future study should include or control the types of online community and users’ needs. Fourth, we did not consider advertising forms on users’ reaction. Social communities can choose advertising forms, e.g., Twitter adopts Promoted Tweets, and Linkedin uses Sponsored Updates, and Inmail. Thus, a following-up research will examine the effect of various advertising forms on user’s responses.

## Author Contributions

The authors worked as a team and made contributions throughout. FZ initiated the research, designed the questionnaire, developed the idea of manuscript, and collected the data. RT conceived the theory, developed the theoretical framework, analyzed the data, and wrote the manuscript. YY supervised and corrected the draft of manuscript. TX offered correction idea, assisted the drafting of revised manuscript. All authors read and approved the final manuscript.

## Conflict of Interest Statement

The authors declare that the research was conducted in the absence of any commercial or financial relationships that could be construed as a potential conflict of interest.
